# A preliminary study of mercury exposure and blood pressure in the Brazilian Amazon

**DOI:** 10.1186/1476-069X-5-29

**Published:** 2006-10-10

**Authors:** Myriam Fillion, Donna Mergler, Carlos José Sousa Passos, Fabrice Larribe, Mélanie Lemire, Jean Rémy Davée Guimarães

**Affiliations:** 1 Centre de recherche interdisciplinaire sur la biologie, la santé, la société et l'environnement (CINBIOSE), Université du Québec à Montréal, C.P. 8888, Succ. Centre-Ville, Montréal, Québec H3C 3P8, Canada; 2 Département de Mathématiques, Université du Québec à Montréal, C.P. 8888, Succ. Centre-Ville, Montréal, Québec H3C 3P8, Canada; 3 Laboratório de Traçadores, Instituto de Biofísica, Universidade Federal do Rio de Janeiro, Bloco G, CCS, Ilha do Fundão, Rio de Janeiro (RJ), CEP 21949-900, Brasil

## Abstract

**Background:**

Fish is considered protective for coronary heart disease (CHD), but mercury (Hg) intake from fish may counterbalance beneficial effects. Although neurotoxic effects of methylmercury (MeHg) are well established, cardiovascular effects are still debated. The objective of the present study was to evaluate blood pressure in relation to Hg exposure and fish consumption among a non-indigenous fish-eating population in the Brazilian Amazon.

**Methods:**

The study was conducted among 251 persons from six communities along the Tapajós River, a major tributary of the Amazon. Data was obtained for socio-demographic information, fish consumption, height and weight to determine body mass index (BMI), systolic and diastolic blood pressure, and Hg concentration in hair samples.

**Results:**

Results showed that overall, systolic and diastolic blood pressure, were relatively low (mean: 113.9 mmHg ± 14.6 and 73.7 mmHg ± 11.0). Blood pressure was significantly associated with hair total Hg (H-Hg), age, BMI and gender. No association was observed between fish consumption and blood pressure, although there were significant inter-community differences. Logistic regression analyses showed that the Odds Ratio (OR) for elevated systolic blood pressure (≥ 130 mmHg) with H-Hg ≥ 10 μg/g was 2.91 [1.26–7.28], taking into account age, BMI, smoking, gender and community.

**Conclusion:**

The findings of this preliminary study add further support for Hg cardiovascular toxicity.

## Background

Mercury (Hg), a worldwide pollutant transported by air and water throughout the planet, poses a particular challenge to global health. On the one hand, Hg is recognized as one of the most dangerous environmental contaminants [1]. On the other hand, fish, a very nutritious food, is the major vehicle for its transmission to humans in its organic form, methylmercury (MeHg). For populations that rely on fish as their main source of protein, this represents an important public health dilemma [2,3], particularly since recent evidence suggests that fish can be both cardioprotective and cardiotoxic, depending upon their contribution to essential fatty acids and Hg body burden [4-8].

Fish is considered a very healthy food because it is rich in proteins, poor in saturated fats, and can be protective for coronary heart disease (CHD) [9,10]. Marine fish oils are rich in omega-3, an essential fatty acid, known to reduce CHD risk [11-14]. Populations that traditionally consume large amounts of marine fish generally experience lower rates of mortality from heart disease [15-18]. But Hg intake from fish may counterbalance beneficial effects [6,7]. Hg exposure has been associated with an increased risk of myocardial infarction, as well as death from cardiovascular disease and all causes [4,19]. Guallar et al. observed both the negative effects of Hg exposure and the positive effects of omega-3's in a case-control study involving a group of men with a first diagnosis of myocardial infarction and a reference group of healthy men; risk for myocardial infarction increased with Hg levels in toenails and decreased with serum omega-3 levels [4]. The United States Health Professionals Follow-up Study did not show an association between Hg and risk for coronary heart disease, but dentists, with elemental Hg exposure, made up 63.6% of controls [20]. Moreover, both of these studies used total Hg concentration in toenails and did not differentiate between organic and inorganic Hg.

Blood pressure, a good indicator of risk for cardiovascular disease, is a parameter relatively easy to measure, even in remote field conditions. Bulliyya et al. showed that fish consumption was associated with lower mean systolic and diastolic blood pressure among older men and women from coastal fishing villages in India [21]. In contrast, an increased incidence of hypertension and cerebrovascular disease has been reported among aging patients with chronic Minamata disease [22]. In the Greenland Inuit population, autopsies revealed that MeHg levels in organs are generally high, and blood pressure levels are similar to those in industrialized countries [23]. Epidemiologic studies relating MeHg and blood pressure have reported inconsistent findings [24,25]. In animal studies, fish proteins lowered blood pressure in spontaneously hypertensive rats [26-28], while in rats that had developed hypertension after sucrose administration, fish oils were able to reverse the alterations on metabolic parameters and blood pressure [29]. However, long term experimental studies suggest that low dose MeHg exposure can lead to irreversible hypertension that remains many months after cessation of exposure [30].

In the Brazilian Amazon, a number of studies have reported high levels of Hg in fish and in humans, and significant relations between fish consumption and bioindicators of Hg exposure [31-38]. Extensive interdisciplinary studies on the source, transmission and Hg contamination and its effects on populations of the Tapajós River have shown that Hg contamination is very widespread in this region and mainly derived from deforestation and 'slash and burn' agricultural practices [39-43]. When erosion drains soil sediments into the waterways, the inorganic Hg, naturally present in these soils, is transformed into MeHg through bacterial activity and enters the aquatic food chain [44-46]. For the traditional populations, living on the banks of the Amazon and its tributaries, fish is the dietary mainstay, with the large majority eating fish daily or several times a week [34-36,38].

Most communities living along the Tapajós River are not indigenous peoples, but traditional communities with mixed ethnic backgrounds [47]. Their diet consists primarily of fish, with manioc, rice, tomatoes, beans, fruit and some meat [36]. Common risk factors for high blood pressure, such as sodium intake, obesity and a sedentary life style are rare. As part of a global, interdisciplinary project on the sources, transmission and effects of Hg in a riverside Amazonian environment (State of Pará, Brazil), we conducted this preliminary study to examine blood pressure parameters with respect to Hg exposure and fish consumption among traditional communities living along the Tapajós River.

## Methods

### Population and sampling

**Figure 1:**
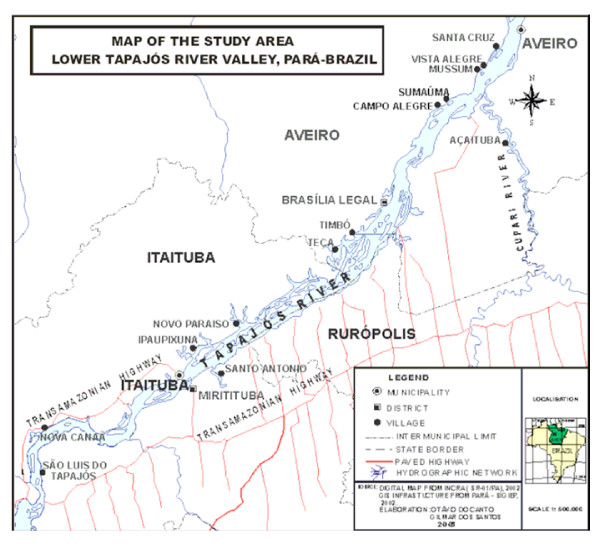
Map of the study area

The study population was from 6 communities (São Luiz do Tapajós, Nova Canaã, Santo Antônio, Mussum, Vista Alegre and Açaituba), living along the Tapajós River, a major tributary of the Amazon River (Figure 1). Since it was not possible to carry out a rigorous random sampling strategy in the conditions of the Amazon, a convenience sample was used and the age and sex distributions were compared to the underlying population, determined through a house-to-house survey. Recruitment into the study was carried out during the house-to-house survey and at village meetings, during which the research project was explained, and villagers were invited to participate on a voluntary basis. Inclusion criteria were persons above 15 years of age and for the present study, those with reported diabetes were excluded.

The study was approved by the Federal University of Rio de Janeiro, which has a mandate from the Ethics Review Board of the Conselho Nacional de Desenvolvimento Científico e Tecnológico (CNPq) of Brazil, and the University of Quebec at Montreal and all participants signed an informed consent form, which was read to them.

### Socio-demographic information

Two trained interviewers met the participants in their villages. All participants went through an interview of approximately one hour. A questionnaire was used to determine the socio-demographic variables: age, education, work and residence history, smoking and drinking habits, as well as fish consumption and medical history. At the end of the interview, all participants were weighed and measured, and body mass index (BMI = weight (kg)/height (m)^2^) was calculated.

### Characterization of fish consumption

A seven-day recall of fish consumption was used. To facilitate the detailing of their fish consumption, a list of the fish species usually consumed in the Tapajós region was prepared [48]. For each of the past seven days, the participants indicated the number of fish meals and the species consumed at each meal. Fish that were not on the list were likewise noted.

### Mercury exposure assessment

Hair has been extensively used as a bioindicator for current and retrospective evaluation of Hg [49]. This non-invasive method provides samples that can be stored for a long time without deterioration before being analyzed. When hair grows, the intense metabolic activity at the follicle level exposes hair to elements present in the blood, including heavy metals [50].

In the present study, hair strands from the root were cut from the occipital region and stored in plastic bags, with the end root stapled. The first centimetre was analyzed for each sample. Hair mercury concentration (H-Hg) was determined by cold vapour atomic absorption spectrometry (CVAAS) after digestion with HNO3, H2SO4 and K2MnO4 and spinning with hydroxylamine chlorhydrate before reading [51]. Hair samples were analyzed in the Laboratório de Traçadores, Centro de Ciências da Saúde, Instituto de Biofísica Carlos Chagas Filho, Universidade Federal do Rio de Janeiro, Brazil. Precision and accuracy of Hg determination were ensured using internal hair standards, provided by the International Atomic Energy Association.

### Blood pressure assessment

The same nurse measured blood pressure throughout the study, after each participant had been sitting for five minutes, relaxed and not moving. Blood pressure was assessed in a seated position, with the arm supported at heart level, and without any tight clothing constricting the arm. The cuff was placed with the centre of the bladder over the brachial artery. Systolic and diastolic pressures, recorded in increments of 1 cmHg (10 mmHg), were assessed using a sphygmomanometer (Blood Pressure Monitor Kit, Mark of Fitness, Model MF-20, Stethoscope attached to cuff). The nurse had no knowledge of participants' Hg exposure, and since blood pressure was assessed prior to administration of the health questionnaire, nor was she aware of the potential risk factors such as smoking and diabetes.

### Statistical analyses

Data was entered and analyzed in the StatView (Version 5.0.1) software (SAS Institute). Descriptive analyses were performed to characterize the population. Multivariate analyses were performed to identify the factors that influenced systolic and diastolic blood pressure. Logistic regression analyses, using categorized data, were used to determine the Odds Ratio (OR) for elevated arterial blood pressure.

## Results

Relevant data were collected from 259 adults (≥ 15 years of age) representing 39.2% of the total adult population. Participants' age and sex distribution were similar to the underlying population (Table 1). The age distribution in the population is typical of that found in developing countries, with about 45 % of the population under 15 years of age (543 people < 15 years from a total population of 1204 persons).

**Table 1:** Age distribution and participation in the study population

Age categories	Total population	# of participants	% participation
15 – 24	217	79	36.4
24 – 34	142	57	40.1
35 – 44	117	56	47.9
45 – 54	84	29	34.5
55 – 64	58	23	39.7
≥ 65	43	15	34.9
Total	661	259	39.2

Of the 259 adults who participated in the study, 7 were excluded from the present analyses for reported diagnosed diabetes, a known risk factor for hypertension and 1 for missing data. The characteristics of the remaining 251 participants are reported in Table 2. Mean age of the study population was 35.2 years (15 to 89 years); 19.9% were over 50. Only 17.5% of the population was overweight, with a BMI above 25 kg/m^2^, and 4% were considered obese (BMI > 30 kg/m^2^). Although 29.8% of the population smoked, the mean number of cigarettes/day was 8.0 (median = 5.0).

**Table 2:** Socio-demographic characteristics of the study population

	Women			Men		
Characteristics	n	Mean ± SD	%	N	Mean ± SD	%

Age (years)	118	34.4 ± 15.3	100	133	35.8 ± 16.2	100
Education (years)	118	4.1 ± 2.6	100	133	3.3 ± 2.5	100
*Alcohol consumption*						
Drinks	27		22.9	67		50.4
No longer drinks	15		12.7	25		18.8
Never drank	76		64.4	41		30.8
*Smoking habits*						
Smokes	25		21.2	50		37.6
No longer smokes	22		18.6	28		21.0
Never smoked	71		60.2	56		41.4
Ever suffered malaria	56		47.5	94		70.7
Body mass index	118	22.5 ± 4.1	100	133	22.2 ± 3.0	100

### Fish consumption

Mean fish consumption was 6.8 ± 4.7 meals in the week preceding the interview, for an average of 1 fish meal/day, with 3.3 ± 3.6 piscivorous fish meals and 3.4 ± 3.2 non-piscivorous fish meals. The five species most commonly consumed were: Aracu (*Shizodon *sp.), Pescada (*Plagioscion *sp.), Tucunaré (*Cichla *sp.), Caratinga (*Geophagus *sp.) and Pacu (*Mylossoma *sp.). Fish consumption did not vary with any of the socio-demographic variables listed in Table 2.

### Mercury exposure

Mean H-Hg was 17.8 μg/g ± 12.0 (0.21 μg/g – 77.2 μg/g) and 69.7% of the participants had H-Hg ≥ 10 μg/g. Multiple regression analysis of the variables that influence H-Hg showed that men had higher levels than women (p = 0.04) and decreased with educational level (p = 0.04). There were significant differences between communities (p < 0.001) and a positive association with consumption of piscivorous fish (β = 0.70; p = 0.002), but not non-piscivorous fish (p = 0.23).

### Blood pressure

Mean systolic pressure was 113.9 mmHg ± 14.6, ranging from 90 mmHg to 170 mmHg and mean diastolic pressure was 73.7 mmHg ± 11.0, ranging from 60 mmHg to 110 mmHg.

Univariate analyses showed that systolic blood pressure was positively associated with age (r^2 ^= 0.12; p < 0.001), higher in men than in women (r^2 ^= 0.04; p = 0.001), positively associated with BMI (r^2 ^= 0.06; p < 0.001), higher among smokers (r^2 ^= 0.03; p = 0.01), and positively associated with H-Hg levels (r^2 ^= 0.02; p = 0.046). There were also borderline significant differences between communities (adjusted r^2 ^= 0.02; p = 0.05). For diastolic blood pressure, age (r^2 ^= 0.15; p < 0.001), sex (r^2 ^= 0.04; p < 0.001), BMI (r^2 ^= 0.08; p < 0.001) and smoking (r^2 ^= 0.03; p < 0.01) were correlated. The relation between H-Hg and diastolic pressure was not significant (p = 0.15).

**Figure 2:**
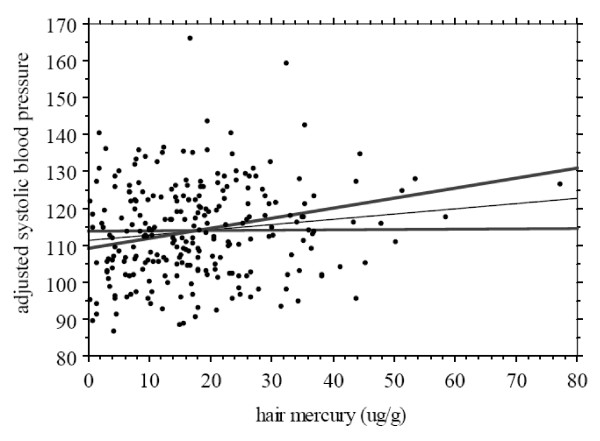
Scatter plot of systolic blood pressure (adjusted for covariates: age, sex, BMI, smoking and community) in relation to mercury exposure; regression line and 95% confidence interval (dotted lines)

Multivariate analyses showed that all of the previous variables, with the exception of smoking entered significantly into the regression model for systolic pressure: adjusted r^2 ^= 0.21; p < 0.001, with H-Hg explaining 1.7% of the total variance (β = 0.14; p = 0.03). For diastolic pressure, only age, sex and community entered significantly into the model. Fish consumption, measured by the total number of fish meals over the seven days previous to the interview, did not enter into the models (p = 0.33). Species-specific analyses with the five most commonly consumed fish (Aracu, Pescada, Tucunaré, Caratinga, Pacu) also showed no influence of fish consumption on systolic and diastolic blood pressures. Having suffered from malaria was not related to H-Hg or to blood pressure. Figure 2 shows a scatter plot of systolic blood pressure, adjusted for the significant covariates, in relation to H-Hg.

Only 8% of the study group suffered from hypertension (systolic pressure ≥ 140 mmHg). Thus, for logistic regression analyses, elevated blood pressure (≥ 130 mm Hg) was used as a cut-off. A total of 52 persons (21.0%) had elevated systolic blood pressure while 42 (16.7%) had elevated diastolic pressure (≥ 90 mm Hg). Elevated systolic pressure was higher in those over 50 years (36.0% vs. 16.9%; Fisher's Exact test: p < 0.01), for those with BMI above 25 kg/m^2 ^(36.4% vs. 17.4%; p = 0.01) and for smokers (29.3% vs. 17.1%; p = 0.04); men had a higher prevalence than women (26.3% vs. 14.4; p = 0.02), as did those with H-Hg levels ≥ 10 μg/g (24.0% vs. 13.2%; p = 0.06). Prevalence of elevated systolic pressure varied between communities (Pearson χ^2^: p = 0.03), with the highest at 35.9% and the lowest at 10.8%. Elevated diastolic pressure was significantly associated with age over 50 years (30.0% vs. 13.4%; Fisher's exact test: p = 0.01), BMI over 25 (29.6% vs. 14.0%; p = 0.02), while differences between men and women showed a tendency (21.1% in men vs. 11.9% in women; p = 0.06) and the difference for H-Hg was not significant (11.8% for H-Hg < 10 μg/g vs. 18.9% for H-Hg ≥ 10 μg/g; p = 0.20). No difference was observed between communities (p = 0.18).

Logistic regression analysis, performed with the independent variables age, sex, BMI, smoking, community and H-Hg showed a significant risk with an Odds Ratio (OR) of 2.91 [1.26–7.28] for elevated systolic pressure with H-Hg ≥ 10 μg/g (Table 3); while elevated diastolic pressure showed a tendency (p = 0.08) (Table 4).

**Table 3:** Odd's Ratios from multivariate logistic regression model for elevated systolic blood pressure (≥ 130 mmHg). Mutual adjustment is included.

Variable	OR	p
Hair mercury (≥ 10 μg/g)	2.91 [1.26–7.28]	0.02
Age (≥ 50 years)	2.35 [1.07–5.13]	0.03
Sex (men/women)	2.27 [1.08–4.98]	0.03
BMI (≥ 25 kg/m^2^)	3.17 [1.35–7.22]	0.01
Smoking (smokers/non)	1.69 [0.81–3.51]	0.15
Community		0.01

**Table 4:** Odd's Ratios from multivariate logistic regression model for elevated diastolic blood pressure (≥ 90 mmHg). Mutual adjustment is included.

Variable	OR	p
Hair mercury (≥ 10 μg/g)	2.29 [0.95–6.06]	0.08
Age (≥ 50 years)	2.18 [0.96–4.83]	0.06
Sex (men/women)	2.16 [0.98–4.97]	0.06
BMI (≥ 25 kg/m^2^)	2.95 [1.23–7.03]	0.01
Smoking (smokers/non)	1.62 [0.75–3.51]	0.22
Community		0.04

## Discussion

This preliminary study shows that, in this population, where fish is a dietary mainstay, blood pressure is relatively low, with only 8% displaying hypertension (systolic pressure ≥ 140 mmHg). However, there is a significant dose-effect relation between Hg exposure and blood pressure. Even at these relatively low levels of blood pressure in a fairly young population, an increase was observed with age, BMI, and men had higher blood pressure as compared to women, confirming that the methods were sensitive enough to detect expected changes. These findings, in a population with minimal risk factors for hypertension and with an elevated environmental Hg exposure, offer strong support to a negative effect of Hg on blood pressure parameters.

Because of low blood pressure, we used 130 mmHg as the threshold for elevated blood pressure, as suggested by "The Seventh Report of the Joint National Committee on Prevention, Detection, Evaluation, and Treatment of High Blood Pressure", whose guidelines indicate that individuals with a systolic blood pressure of 120 to 139 mmHg or a diastolic blood pressure of 80 to 89 mmHg should be considered as pre-hypertensive [52]. Others have suggested that the threshold of systolic blood pressure of 130 mmHg is a limit for "borderline hypertension" [53]. In the present study, those with hair Hg levels of 10 μg/g or more had over twice the risk of presenting systolic blood pressure of at least 130 mmHg.

A positive relation between Hg and blood pressure has been reported in animal studies [30], but human studies are less consistent [20]. In a recent study, Pedersen et al. [8] measured blood Hg and blood pressure among four groups of healthy subjects: 1) Danes living in Denmark consuming European food; 2) Greenlanders living in Denmark consuming European food; 3) Greenlanders living in Greenland consuming European food; 4) Greenlanders living in Greenland consuming mainly traditional Greenlandic food. They reported higher blood Hg in Greenlanders as compared to Danes, which they attributed to their higher fish consumption. Pulse pressure was higher and diastolic blood pressure lower in Greenlanders than Danes and blood Hg was positively correlated to pulse pressure. Another study showed that in middle-aged Finnish men, Hg accumulation in the body was associated with accelerated progression of carotid atherosclerosis [19]. In that study, the strongest predictors of the progression of atherosclerosis were elevated systolic blood pressure, high H-Hg content, treatment for dyslipidemia, high dietary intake of iron, cigarette smoking, and old age. The authors suggest that Hg may be a major environmental risk factor for atherosclerosis in humans, even at subtoxic levels, which have not been previously recognized as harmful.

Several studies have reported associations between prenatal exposure to MeHg and cardiovascular functions; however, manifestations vary from one study to another. Although a study on patients with foetal Minamata disease showed that parasympathetic nervous dysfunction might exist in these patients, they did not show elevated blood pressure [55]. Sørensen et al. [56] reported increased systolic and diastolic blood pressures among the Faroese 7 year olds, born from mothers without hypertension, in relation to prenatal MeHg exposure. At 14 years of age, there was no relation with blood pressure, although MeHg exposure was associated with decreased sympathetic and parasympathetic modulation of heart rate variability [57].

Dórea et al. [25] reported a trend of lower increase in blood pressure with age among the higher fish consumers in the Amazonian region, but had no direct measure of fish consumption or of Hg levels. Although in the present study, we did not observe a dose-response relation between fish consumption and blood pressure, the relatively low blood pressure observed in the Tapajós riverine villagers may be related to their general diet of fish, coupled to other lifestyle factors. Some fish may have more of a positive effect than others, but this was not apparent in the present study, which relied on the number of fish meals over the past seven days as an indicator of fish eating habits. Cardio-protection has been reported in relation to omega-3 [15-17,58] and to fish consumption [10]. However, studies of freshwater sports fishers showed no relation between fish consumption, omega-3 levels in blood and/or blood pressure [59,60]. Freshwater fish have lower levels of omega-3 fatty acids compared to marine fish [2,61], which might explain the lack of relation between fish consumption and blood pressure in this study and others, but no data on fish omega-3 levels exist for Amazonian fish.

Studies have shown that fish cooking methods, such as deep frying and surface frying with oil, changes the lipid and moisture content as well as the fatty acid composition of fish products [62,63]. Deep-frying Baltic herring (*Clupea harengus membras*) in rapeseed oil changed the fatty acid compositions to that of the frying oil, increased the amounts of monounsaturated fatty acids and omega-6, while decreasing the levels of omega-3 [62]. Better fatty acid ratios have been observed for roasted salmon compared to fried samples; roasting did not modify the fat content, whereas frying increased the fat content 2-fold [63]. In the Tapajós region, fish is mainly fried or boiled, which possibly influences the fatty acid composition.

Another possible explanation of low blood pressure levels in this population could be low sodium intake. There are few prepared foods with high sodium content in these villages and salt intake comes principally from salting food. Further studies on cardiovascular function in these communities should assess urinary sodium concentrations.

There are several limitations to this preliminary study. First, a convenience sample was used. Although data collection on convenience samples has been shown to appropriately represent the underlying population in other settings [64,65], this sampling strategy may have introduced some selection bias in the present study. We did, however, achieve a participation rate of 35% in this adult population, well represented in most age categories. Second, although persons did relax quietly for 5 minutes prior to taking blood pressure, only a single measure was used. This would, however, increase the variability of the response and tend to minimize any relation with Hg exposure.

In the Brazilian Amazon, our research group is working with local populations to identify factors that influence Hg uptake and metabolism in fish and humans in order maximize the nutritional intake from fish and minimize toxic risk. This is an appropriate region to carry out case control studies to further knowledge on the positive and negative effects of fish consumption and Hg exposure on cardiovascular health. More extensive studies on cardiovascular parameters, including R-R variability and the role of fatty acids are currently under way.

## Abbreviations

BMI – body mass index

CHD – coronary heart disease

Hg – mercury

H-Hg – hair total mercury

MeHg – methylmercury

OR – Odds Ratio

## Competing interests

The author(s) declare that they have no competing interests.

## Authors' contributions

MF is a doctoral student. She participated in the fieldwork, data entry and analysis and writing of the present paper. DM is principal investigator of this study. As such, she participated in the design and the planning, data analysis and writing of the present paper. MF and DM contributed equally to this work. CJSP is a doctoral student. He co-ordinated the fieldwork and the laboratory analysis for hair mercury levels and participated in the data entry. FL is a professor in the mathematics department of Université du Québec à Montréal. He supervised the statistical analysis. ML is a doctoral student who participated in the preparation and realisation of the fieldwork. JRDG is a co-investigator in the study. He supervised the fieldwork and the laboratory analyses for hair mercury. All authors read and approved the final manuscript.

## References

[B1] Watanabe C, Satoh H (1996). Evolution of our understanding of methylmercury as a health threat. Environ Health Perspec.

[B2] Mahaffey KR (2004). Fish and shellfish as dietary sources of methylmercury and the omega-3 fatty acids, eicosahexaenoic acid and docosahexaenoic acid: risks and benefits. Environ Res.

[B3] Clarkson TW, Magos L, Myers GJ (2003). The toxicology of mercury current exposures and clinical manifestations. N Engl J Med.

[B4] Guallar E, Sanz-Gallardo I, Van't Veer P, Bode P, Aro A, Gómez-Aracena J, Kark JD, Riemersma RA, Martín-Moreno JM, Kok FJ (2002). Mercury, fish oils, and the risk of myocardial infarction. N Engl J Med.

[B5] Rissanen T, Voutilainen S, Nyyssönen K, Lakka TA, Salonen JT (2000). Fish oil-derived fatty acids, docosahexaenoic acid and docosapentaenoic acid, and the risk of acute coronary events – The Kuopio Ischaemic Heart Disease Risk Factor Study. Circ.

[B6] Chan HM, Egeland GM (2004). Fish Consumption, Mercury Exposure, and Heart Diseases. Nutr Rev.

[B7] Stern AH (2005). A review of the studies of the cardiovascular health effects of methylmercury with consideration of their suitability for risk assessment. Environ Res.

[B8] Pedersen EB, Jørgensen ME, Pedersen MB, Siggaard C, Sørensen TB, Mulvad G, Hansen JC, Asmund G, Skjoldborg H (2005). Relationship between mercury in blood and 240h ambulatory blood pressure in Greenlanders and Danes. AJH.

[B9] Millen BE, Quatromoni PA (2001). Nutritional research within the Framingham Heart Study. J Nutr Health Aging.

[B10] Whelton SP, He J, Whelton PK, Muntner P (2004). Meta-analysis of observational studies on fish intake and coronary heart disease. Am J Cardiol.

[B11] Kris-Etherton PM, Harris WS, Appel LJ, for the AHA Nutrition Committee (2003). Omega-3 fatty acids and cardiovascular disease: New recommendations from the American Heart Association. Arterioscler Thromb Vasc Biol.

[B12] Kris-Etherton PM, Harris WS, Appel LJ, for the Nutrition Committee (2003). Fish consumption, fish oil, omega-3 fatty acids, and cardiovascular disease. Arterioscler Thromb Vasc Biol.

[B13] Sinclair R (2000). Good, bad or essential fats: what is the story with Omega-3?. Nutr Food Sci.

[B14] Calder PC (2004). n-3 Fatty acids and cardiovascular disease: evidence explained and mechanisms explored. Clin Sci (Lond).

[B15] Dewailly E, Blanchet C, Gingras S, Lemieux S, Sauvé L, Bergeron J, Holub BJ (2001). Relations between n-3 fatty acid status and cardiovascular disease risk factors among Quebecers. Am J Clin Nutr.

[B16] Dewailly E, Blanchet C, Gingras S, Lemieux S, Holub BJ (2002). Cardiovascular disease risk factors and n-3 fatty acid status in the adult population of James Bay Cree. Am J Clin Nutr.

[B17] Dewailly E, Blanchet C, Gingras S, Lemieux S, Holub BJ (2003). Fish consumption and blood lipids in three ethnic groups of Quebec (Canada). Lipids.

[B18] Oomen CM, Feskens EJ, Rasanen L, Fidanza F, Nissinen AM, Menotti A, Kok FJ, Kromhout D (2000). Fish consumption and coronary heart disease mortality in Finland, Italy, and The Netherlands. Am J Epidemiol.

[B19] Salonen J, Seppänen K, Nyyssönen K, Korpela H, Kauhanen J, Kantola M, Tuomilehto J, Esterbauer H, Tatzber F, Salonen R (1995). Intake of mercury from fish, lipid peroxidation, and the risk of myocardial infarction and coronary, cardiovascular, and any death in Eastern Finnish men. Circ.

[B20] Yoshizawa K, Rimm EB, Morris JS, Spate VL, Hsieh C-C, Spiegelman D, Stampfer MJ, Willett WC (2002). Mercury and the risk of coronary heart disease in men. New Engl J Med.

[B21] Bulliyya G, Reddy PC, Reddanna P (1999). Arterial pressures in fish-consuming and non-fish-consuming populations of coastal south India. Asia Pacific J Clin Nutr.

[B22] Uchino M, Tanaka Y, Ando Y, Yonehara T, Hara A, Mishima I, Okajima T, Ando M (1995). Neurological features of chronic Minamata disease (organic mercury poisoning) and incidence of complications with aging. J Environ Sci Health.

[B23] Mulvad G, Pederson HS, Hansen JC, Dewailly E, Jul E, Pederson M, Deguchi Y, Newman WP, Malcom GT, Tracy RE, Middaugh JP, Bjerregaard P (1996). The Inuit diet. Fatty acids and antioxidants, their role in ischemic heart disease, and exposure to organochlorines and heavy metals. An international study. Arctic Med Res.

[B24] Vupputuri S, Longnecker MP, Daniels JL, Guo X, Sandler DP (2005). Blood mercury level and blood pressure among US women: results from the National Health and Nutrition Examination Survey 1999–2000. Environ Res.

[B25] Dórea GD, de Souza JR, Rodrigues P, Ferrari I, Barbosa AC (2005). Hair mercury (signature of fish consumption) and cardiovascular risk in Munduruku and Kayabi Indians of Amazonia. Environ Res.

[B26] Aguila MB, Sa Silva SP, Pinheiro AR, Mandarim-de-Lacerda CA (2004). Effects of long-term intake of edible oils on hypertension and myocardial and aortic remodelling in spontaneously hypertensive rats. J Hypertension.

[B27] Ait-Yahia D, Madani S, Savelli J-L, Prost J, Bouchenak M, Belleville J (2003). Dietary fish protein lowers blood pressure and alters tissue polyunsturated fatty acid composition in spontaneously hypertensive rats. Nutr.

[B28] Ait-Yahia D, Madani S, Prost J, Bouchenak M, Belleville J (2004). Fish protein improves blood pressure but alters HDL_2 _and HDL_3 _composition and tissue lipoprotein lipase activities in spontaneously hypertensive rats. Eur J Nutr.

[B29] Aguilera AA, Díaz GH, Barcelata ML, Guerrero OA, Ros RMO (2004). Effects of fish oil on hypertension, plasma lipids, and tumor necrosis factor-α in rats with sucrose-induced metabolic syndrome. J Nutr Biochem.

[B30] Wakita Y (1987). Hypertension induced by methyl mercury in rats. Toxicol Appl Pharmacol.

[B31] Malm O, Branches FJ, Akagi H, Castro MB, Pfeiffer WC, Harada M, Bastos WR, Kato H (1995). Mercury and methylmercury in fish and human hair from the Tapajos river basin, Brazil. Sci Total Environ.

[B32] Dolbec J, Mergler D, Sousa Passos CJ, Sousa de Morais S, Lebel J (2000). Methylmercury exposure affects motor performance of a riverine population of the Tapajos River, Brazilian Amazon. Int Arch Occup Environ Health.

[B33] Sampaio da Silva D, Lucotte M, Roulet M, Poirier H, Mergler D, de Oliveira Santos E, Crossa M (2005). Trophic structure and bioaccumulation of mercury in fish of 3 natural lakes of the Brazilian Amazon. Water, Air Soil Pollut.

[B34] Lebel J, Roulet M, Mergler D, Lucotte M, Larribe F (1997). Fish diet and mercury exposure in a rivarine Amazonian population. Water Air Soil Pollut.

[B35] Dolbec J, Mergler D, Larribe F, Roulet M, Lebel J, Lucotte M (2001). Sequential analysis of hair mercury levels in relation to fish diet of an Amazonian population, Brazil. Sci Tot Environ.

[B36] Passos CJ, Mergler D, Gaspar E, Morais S, Lucotte M, Larribe F, Davidson R, de Grosbois S (2003). Eating tropical fruits reduces mercury exposure from fish consumption in the Brazilian Amazon. Environ Res.

[B37] Dorea JG, Barbosa AC, Ferrari I, de Souza (2005). Fish consumption (hair mercury) and nutritional status of Amazonian Amer-Indian children. Amer J Human Biol.

[B38] Hacon S, Yokoo E, Valente J, Campos RC, da Silva VA, de Menezes AC, de Moraes LP, Ignotti E (2000). Exposure to mercury in pregnant women from Alta Floresta – Amazon basin, Brazil. Environ Res.

[B39] Roulet M, Lucotte M, Farella N, Serique G, Coelho H, Passos CJS, de Jesus da Silva E, de Andrade PS, Mergler D, Guimarães JR, Amorim M (1999). Effects of recent human colonization on the presence of mercury in Amazonian ecosystems. Water Air Soil Pollut.

[B40] Roulet M, Lucotte M, Canuel R, Farella N, Courcelles M, Guimarães JRD, Mergler D, Amorim M (2000). Increase in mercury contamination recorded in lacustrine sediments following deforestation in the central Amazon. Chem Geol.

[B41] Roulet M, Guimarães JRD, Lucotte M (2001). Methylmercury production and accumulation in sediments and soils of an Amazonian floodplain – Effect of seasonal inundation?. Water Air Soil Pollut.

[B42] Roulet M, Lucotte M, Canuel R, Farella N (2001). Spatio-temporal geochemistry of mercury in waters of the Tapajós and Amazon rivers, Brazil. Limno Oceanogr.

[B43] Farella N, Lucotte M, Louchouarn P, Roulet M (2001). Deforestation modifying terrestrial organic transport in the Rio Tapajós, Brazilian Amazon. Org Geochem.

[B44] Spry DJ, Wiener JG (1991). Metal bioavailability and toxicity to fish in low-alkalinity lakes: a critical review. Environ Pollut.

[B45] Guimarães JR, Meili M, Hylander LD, de Castro e Silva E, Roulet M, Mauro JB, de Lemos R (2000). Mercury net methylation in five tropical flood plain regions of Brazil: high in the root zone of floating macrophyte mats but low in surface sediments and flooded soils. Sci Tot Environ.

[B46] Guimarães JR, Roulet M, Lucotte M, Mergler D (2000). Mercury methylation along a lake-forest transect in the Tapajós river floodplain, Brazilian Amazon: seasonal and vertical variations. Sci Tot Environ.

[B47] Murrieta RSS (2001). Dialética do sabor: alimentação, ecologia e vida cotidiana em comunidades ribeirinhas da Ilha de Ituqui, Baixo Amazonas, Pará. Revista Antropol São Paulo, USP.

[B48] Passos CJ, Mergler D, Gaspar E, Morais S, Lucotte M, Larribe F, de Grosbois S (2001). General characterization of the diet of a riverside population in the Brazilian Amazon. Rev Saude Ambiente.

[B49] National Research Council (2000). Toxicological Effects of Methylmercury.

[B50] Katz SA, Katz RB (1992). Use of hair analysis for evaluating mercury intoxication of the human body: a review. J Appl Toxicol.

[B51] Bastos WR, Malm O, Pfeiffer WC, Cleary D (1998). Establishment and analytical quality control of laboratories for Hg determination in biological and geological samples in the Amazon-Brazil. Ciência e Cultura.

[B52] Chobanian AV, Bakris GL, Black HR, Cushman WC, Green LA, Izzo JL, Jones DW, Materson BJ, Oparil S, Wright JT, Roccella EJ, Joint National Committee on Prevention, Detection, Evaluation, and Treatment of High Blood Pressure. National Heart, Lung, and Blood Institute; National High Blood Pressure Education Program Coordinating Committee (2003). Seventh report of the Joint National Committee on Prevention, Detection, Evaluation, and Treatment of High Blood Pressure. Hypertension.

[B53] Mainous AG, Everett CJ, Liszka H, King DE, Egan BM (2004). Prehypertension and Mortality in a Nationally Representative Cohort. Am J Cardiol.

[B54] Salonen JT, Seppänen K, Lakka TA, Salonen R, Kaplan GA (2000). Mercury accumulation and accelerated progression of carotid atherosclerosis: a population-based prospective 4-year follow-up study in men in eastern Finland. Atherosclerosis.

[B55] Oka T, Matsukura M, Okamoto M, Harada N, Kitano T, Miike T, Futatsuka M (2002). Autonomic nervous functions in fetal-type Minamata disease patients: assessment of heart rate variability. Tohoku J Exp Med.

[B56] Sørensen N, Murata K, Budtz-Jørgensen E, Weihe P, Grandjean P (1999). Prenatal methylmercury exposure as a cardiovascular risk factor at seven years of age. Epidemiol.

[B57] Grandjean P, Murata K, Budtz-Jørgensen E, Weihe P (2004). Cardiac autonomic activity in methylmercury neurotoxicity: 14-year follow-up of a Faroese birth cohort. J Pediatrics.

[B58] Dewailly E, Blanchet C, Lemieux S, Sauvé L, Gingras S, Ayotte P, Holub BJ (2001). n-3 fatty acids and cardiovascular disease risk factors among the Inuit in Nunavik. Am J Clin Nutr.

[B59] Godin C, Shatenstein B, Paradis G, Kosatsky T (2003). Absence of cardiovascular benefits and sportfish consumption among St- Lawrence River angler. Environ Res.

[B60] Philibert A, Vanier C, Abdelouahab N, Chan HM, Mergler D (2006). Relationship between fish intake and serum fatty acid profiles: a community-based study in Quebec, Canada. J Clin Nutr.

[B61] Innis SM, Rioux FM, Auestad N, Ackman RG (1995). Marine and freshwater fish oil varying in arachidonic, eicosapentaenoic and docosahexaenoic acids differ in their effects on organ lipids and fatty acids in growing rats. J Nutr.

[B62] Aro T, Tahvonen R, Mattila T, Nurmi J, Sivonen T, Kallio H (2000). Effects of season and processing on oil content and fatty acids of Baltic herring (Clupea harengus membras). J Agric Food Chem.

[B63] Echarte M, Zulet MA, Astiasaran I (2001). Oxidation process affecting fatty acids and cholesterol in fried and roasted salmon. J Agric Food Chem.

[B64] Kelly H, Riddell MA, Gidding HF, Nolan T, Gilbert GL (2002). A random cluster survey and a convenience sample give comparable estimates of immunity to vaccine preventable diseases in children of school age in Victoria, Australia. Vaccine.

[B65] Zelinski EM, Burnight KP, Lane CJ (2001). The Relationship Between Subjective and Objective Memory in the Oldest Old: Comparisons of Findings From a Representative and a Convenience Sample. J Aging and Health.

